# The Relationships between Physical Activity, Exercise, and Sport on the Immune System

**DOI:** 10.3390/ijerph19116777

**Published:** 2022-06-01

**Authors:** Pedro Forte, Luís Branquinho, Ricardo Ferraz

**Affiliations:** 1 Department of Sports, Higher Institute of Educational Sciences of the Douro, 4560-708 Penafiel, Portugal; luis.branquinho@iscedouro.pt; 2 Department of Sport Sciences and Physical Education, Instituto Politécnico de Bragança (IPB), 5300-253 Bragança, Portugal; ricardompferraz@gmail.com; 3 Research Centre in Sports Sciences, Health and Human Development (CIDESD), 6201-001 Covilhã, Portugal; 4 CI-ISCE, ISCE Douro, 4560-708 Penafiel, Portugal; 5 Department of Sports Sciences, University of Beira Interior, 6201-001 Covilhã, Portugal

## 1. Introduction

During their lifetime, human beings are exposed to different microorganisms (i.e., virous, bacteria, fungi, and germs), parasites, and cancerous cells. This exposure endows the immunological system with the ability to differentiate what may be accepted or rejected by the human body, which in turn is controlled byantigens and antibodies. Antigens are any substance that the human immune system may recognize to stimulate an immunological response while antibodies are B-cell proteins responsible for identifying and marking the invader agent (i.e., antigen) to be neutralized or eliminated by the immune system. Exposure to different pathogenic agents may have two different outcomes: the exposure may result in improving the immunological resistance, or the exposure may increase the inflammatory response.

With ageing, lifestyle will play an important role in the responsiveness of the human immunological system. Sedentary behavior and excessive intake of hypercaloric energy will inevitably result in higher fat levels. Additionally, several related diseases are prone to appear during the life course (i.e., cardiovascular and respiratory diseases, diabetes, cholesterol, hypertension, chronic inflammation, cancer). This will result in a public health issue, where health-related problems are associated with the human body’s lower capacity to mobilize an efficient immunological response.

Exercise-based therapies helps to improve the immune system, both reducing oxidative stress related to inflammatory-marker levels and improving the body’s immunological response. In addition, these therapies improve physical fitness, and they are acknowledged as an important therapeutic coadjuvant in different diseases. Moreover, there is an association between physical activity, immunological response, and health. However, more studies are required to bolster this evidence.

## 2. Changed Gene Expression, Exercise and Immune System

Changed gene expression (i.e., epigenetics) is multifactorial and depends on environmental conditions, exposure to different pathogenesis agents, and behaviors, such as diet and exercise [1,2,3]. The epigenetics involve changes in genes’ activity without any alteration in DNA sequence. Various stimuli associated with muscular contraction (exercise related) appear to cause changes in skeletal-muscle gene expression. Several signalling kinases respond to these stimuli, suggesting that epigenesis pathways might affect gene expression [4]. The changed gene expression, after twenty weeks of diet and exercise, seems to contribute to change the abdominal adipose tissue and related gene-expression changes in obese women [3]. Furthermore, according to Connolly et al. [5], a single session of intensive physical activity significantly changes the expression of peripheral blood mononuclear cells (i.e., lymphocytes, monocytes, erythrocytes, platelets, and granulocytes). This change is primarily defined by the rapid activation and deactivation of genes involved in stress, inflammation, and tissue repair. For these reasons, exercise-based therapies may contribute to altered gene expression and improved immunological responses.

So far, the evidence shows that acute exercise based on recommendations of different intensities and durations of the ACSM has a significantly negative impact on the immune function of individual T- and B-lymphocytes and neutrophils [6]. The link between intense exercise, oxidative stress, the antioxidant defense systems, and cellular immunological response is well understood [7]. Vider et al. [8] presented that high-intensity exercise caused a reduction in the percentage of activated lymphocyte subsets (CD4+ and CD8+) expressing CD69 in young trained males. They also discovered a reduction in lymphocyte mitogenesis to ConA and PHA, as well as an increase in lipid peroxidation, total antioxidant status (TAS), and catalase activity promptly after exercise.

The goal of sports medicine and the role of physical activity for health, regarding the immune system, is to prevent illnesses and infections. Exercise at high intensity appears to cause oxidative damage and an immediate immunological response. However, understanding the interplay between antioxidant processes and the immune system as a consequence of physical exercise remains an essential and current research field [5]. High-intensity physical activity stimulates oxygen consumption and the formation of reactive oxygen species (ROS). ROS, which are generally reactive molecules and free radicals produced by aerobic respiration and mitochondrial activity, have the potential to be damaging to the human body [8,9]. The imbalance between ROS generation and the antioxidant response of the cell causes oxidative stress. Thus, the greater the oxidative stress, the larger the creation of ROS and the poorer the capacity of cells to react to antioxidants. There is also a link between oxidative stress and inflammatory indicators in the human body [10]. As a result, inflammatory indicators are likely to rise in response to oxidative stress.

Regarding the above mentioned, the importance of physical exercise in the management of oxidative stress and, as a result, the reduction of the inflammatory response becomes evident. Serum total antioxidant status (which correlates to the ability to eliminate free radicals) considerably increases thirty minutes after a session of high-intensity exercise. However, the effects of physical exercise on immunological responses are reliant on a number of variables, such as metabolism, oxidative stress, and changed gene expression as a result of adaptations to physical activity [7].

Overall, it is possible to argue that exercise effects on gene expression may be related to a higher and more efficient adaptation of the genes to control stress, inflammation, and repair tissues. The capacity to minimize these effects may result in an efficient function of the immunological system. Additionally, reducing inflammation ensures a better immunological response. However, more studies are required to deepen the current understanding of exercise epigenetics.

## 3. Ageing, Exercise and Immune System

It is well known that physical activity and exercise play an important role in the independence and general well-being of the elderly. However, ageing is characterized by several changes in people, such as functional fitness decreasing, changes in body compositions (i.e., increasing fat levels and decreasing muscular mass), appearance of physiological diseases (i.e., such as the metabolic syndrome and its risk factors), and biochemical changes (higher inflammatory levels).

However, it is important to know that the physiological impact of ageing will affect the immune system. Typically, the ageing process is related to immunosenescence (the gradual decline in immune function as a consequence of the normal aging process) [11]. Immunosenescence is multifactorial and typically the result of lifelong exposure to different pathogens. This exposure will result in exhaustion of the cellular components of immunity, such as T-Cells and B-Cells [12]. This results in a decrease in resting immunological activity, increasing the risk of infection, tumor growth, and auto-immune illness [13].

The “Immune Risk Profile” is a cluster of human biomarkers that enable the assessment of the immune system as well as the determination of the prognostics of death risk [14,15]. The biomarkers of a weakened immune system are: an inverted ratio of CD4:CD8 T-cell, the mitogen’s declined proliferative T-cell responses, low production levels of the interleukin-2; a high ratio of late- (CD27−/CD28−) to early- (CD27+/CD28+) stage differentiated CD8+ T-cells, an increased proportion of T-cells expressing markers associated with senescence (KLRG1, CD57), and a latent cytomegalovirus infection [16]. This contributes to poorer immune responses and the increased incidence of infection and malignancy seen in the elderly [13].

The ageing process has been explained by the oxidative-stress theory. As oxidative stress increases, immune response lessens. This is true in many biomarkers; however, the T-Cells are the most compromised, possibly due the involution of the thymus gland, a result of ageing. Defects in T-Cell proliferative capacity/responsiveness, interleukin-2 production and receptor expression, signal transduction, and cytotoxicity are the most common biomarkers related to immunosenescence [16].

Some studies show that exercise can have a positive or negative impact on the immune system, depending of the training type, intensity, and duration [17,18,19]. Moderate exercise, appears to help offset the negative effects of ageing on the immune system. However, there have been few studies that compare the immunological responses of young and elderly people to acute and chronic effects of exercise [13]. Moderate exercise appears to help reverse the detrimental effects of ageing on the immune system. The responses of natural killer (NK) cells (those responsible for fighting viral infections and tumor cells) in the elderly are similar to those of younger people, although this may be due to a lower initial proliferative capacity. The activity of NK cells in resting elderly people appears to increase with exercise. However, ageing is associated with an increase in the likelihood of overtraining, which raises the risks of injury [13]. Khammassi et al. [17] compared the effects of two high-intensity interval-training sessions to those of moderate-intensity continuous-training sessions. For the study, sixteen participants were randomly divided into two groups and evaluated after nine weeks. The two groups improved the cardiorespiratory fitness (i.e., VO_2_ max). However, the counts of Leukocyte, lymphocyte, neutrophil, and monocyte presented better results in the moderate-intensity continuous-training group; these results show better outcomes for the participants’ immune function.

## 4. Different Pathologies, Exercise and Immune System

Given the multifactorial dependency of the immune system, it is possible for studies to include pathogenic agents (i.e., virus, bacterias, and others), metabolic illnesses (such as metabolic syndrome and the risk factors), and hormonal dysregulation. Recently, COVID-19 has increased public-health concerns. Metabolic syndrome is one of the major results of sedentarism, typically in developed countries. Finally, in terms of diseases, cancer has been one of the most important topics in medical research aiming to create or find new treatments, medications, and strategies to reduce cancer illness. That said, this section calls for the awareness of the importance of new evidence-based research on COVID-19, metabolic syndrome and its attendant risk factors, cancer, and the importance of exercise-based therapies.

### 4.1. COVID-19

COVID-19 is still a new topic of disease research, and there is a need for a better understanding of its acute and chronic effects, mainly in the cardiorespiratory system, including pulmonary functions. There are some studies assessing post-mortem pulmonary anatomy in patients with and without COVID-19 [20,21,22]. One study reported that, in the deceased, there are two distinctive immunopathological patterns: one presented high local expression of interferon stimulated genes (ISG) and cytokines, high viral loads and limited pulmonary damage; the other pattern shows severely damaged lungs, low ISGs (ISG), low viral loads, and abundant infiltrating activated CD8+ T-cells and macrophages [23]. The ISGs, Cytokines, and CD8+ T are typically responsible for immune responses, pathogenesis resistance and controlling, identification, destroying cells, as well as consuming and digesting viruses [20,21,22]. In another study, postmortem patients with COVID-19 presented higher severe lung vascular (endothelial) injury with a high prevalence of T-cells, thrombosis, and microangiopathy [20]. Some postmortem patients had expressive alveolar damage and massive capillary congestion by microthrombi [21]. Moreover, COVID-19 has been associated with myocarditis, cardiac arrest, and acute heart failure. In summary, COVID-19 disease is related to the impairment of lung activity and cardiorespiratory function; the disease’s main cause of death is respiratory failure [20,21,22]. Therefore, physical activity and exercise programs can be an important therapy to improve cardiorespiratory and vascular function, reducing inflammation and improving the body’s immunological response to the disease.

### 4.2. Metabolic Syndrome

Metabolic syndrome is a public health concern defined by a cluster of major risk factors, such as central obesity, dysglycemia, dyslipidemia, and hypertension [23,24]. For several years, studies have reported that the combination of these factors increases the risk of some diseases, such as atherosclerotic cardiovascular diseases, diabetes mellitus (Type 2), neurological disorders, and cancers [23,25,26].

The most significant metabolically active adipose tissue is visceral adipose tissue, with various endocrine, metabolic, and immunological features for the etiology of metabolic dysfunction illnesses [27]. Anthropometric assessments of visceral fat (i.e., waist circumference) are thought to be a necessary component of diagnosing metabolic syndrome and obesity [27]. Adipose tissue secretes pro-inflammatory cytokines, adipokines, and sex hormones, creating a milieu of pro-inflammatory insulin resistance. The prevalence of metabolic syndrome has been debated, and insulin resistance, together with two other risk factors, is a fundamental and required component [28,29,30]. When excess abdominal adiposity was identified as a major component of the metabolic syndrome, the best cutoff thresholds for defining central adiposity for each community and ethnicity were debated [30]. In this approach, metabolic disorders and body fat create an inflammatory milieu that hampers the immune system’s efficient functioning.

The exercise intervention can help change the body composition, reducing body fat and, therefore, the body’s inflammatory status. Additionally, exercise programs enable the control of different risk factors for metabolic syndrome, reducing the chances of unhealthy inflammation. Overall, exercise-based therapy will be an important and efficient treatment for metabolic syndrome, obesity, and inflammation.

### 4.3. Cancer

Metabolic syndrome, low levels of physical fitness, and unhealthy habits and lifestyles (i.e., sedentary lifestyle) all contribute to increased body fat. Furthermore, the adipose tissue, specifically visceral adipose tissue, secretes several proteins and hormones (typically, adipokines, such as leptin and adiponectin), from their activity, contributing to carcinogenesis [31,32,33,34]. Obesity also results in chronic inflammation, characterized by pro-inflammatory cytokines and acute phase reactants, creating a pro-tumor inflammatory environment, which is ideal for cancer cells to proliferate, grow, and develop [35]. Now, increases in inflammatory markers are associated with higher mortality after cancer. Finally, insulin resistance is one of the main components of the metabolic syndrome and is associated with an increased risk of developing cancer, due to the mitogenic effect of insulin, for example, on breast cells and specifically in breast cancer [28,29,30]. Moreover, an increase in inflammatory biomarkers has been associated with a higher risk of cancer.

Furmaniak et al. [35] found that aerobic and strength training, alone or in combination, was helpful in reducing tiredness. They also found that aerobic exercise improves cardiorespiratory fitness more than strength training, while strength training improves muscle strength, lean body mass, and self-esteem. Changing body composition, generally lowering fat percentage, and increasing fitness levels will help the immune system work better.

## 5. Research Topics

Improving physical fitness by physical activity or exercise programs may change body composition, especially fat levels. As fat levels decrease, the possibility of acquiring new health-related problems also decreases, problems associated with metabolic-syndrome risk factors. As more health issues (i.e., hypertension, diabetes, obesity, cholesterol, virous and bacteria related diseases) develop, the body’s inflammatory response increases. Moreover, increased inflammation compromises the immune system and leads to new health-related problems such as cancer. For these reasons, evidence-based practices are important, especially those that examine the role played by physical activity and exercise therapeutics in reducing inflammation and improving the immune system.
